# Halide lead perovskites for ionizing radiation detection

**DOI:** 10.1038/s41467-019-08981-w

**Published:** 2019-03-06

**Authors:** Haotong Wei, Jinsong Huang

**Affiliations:** Department of Applied Physical Sciences, University of North Carolina, Chapel Hill, NC 27599 USA

## Abstract

Halide lead perovskites have attracted increasing attention in recent years for ionizing radiation detection due to their strong stopping power, defect-tolerance, large mobility-lifetime (*μτ*) product, tunable bandgap and simple single crystal growth from low-cost solution processes. In this review, we start with the requirement of material properties for high performance ionizing radiation detection based on direct detection mechanisms for applications in X-ray imaging and γ-ray energy spectroscopy. By comparing the performances of halide perovskites radiation detectors with current state-of-the-art ionizing radiation detectors, we show the promising features and challenges of halide perovskites as promising radiation detectors.

## Abstract (web summary)

Halide lead perovskites have emerged recently as possible candidates for high performance radiation detectors besides efficient solar cells. Here Wei et al. review the recent progress on perovskite based radiation detectors and suggest that they may compete with the conventional counterparts.

## Introduction

The strong penetrating capability of high-energy ionizing radiation, including X-ray (photon with wavelength from 0.01 to 10 nm) or gamma-ray (γ-ray, photon with wavelength shorter than 0.01 nm), make them attractive in non-destructive probing of inside information of condensed matter subjects. After their interaction with electrons in the atoms to ionize the materials, X-rays and γ-rays carry information of the subjects in their variation of the photon intensity (dose rate), direction, phase, and energy. The detection of the X- and γ-ray variation in good faith thus allows accurate retrieval of the subject information. This enables the broad applications of X-ray in many fields including medical imaging, safety screening, shipping container inspection, defect inspection and quality control in material industry, and contamination detection and quality control inspection in food industry, where X-rays penetrate subjects and its attenuation is recorded^1–5^. The image contrast comes from the difference in absorption or stopping capability of different human tissues or subjects to X-rays. In scientific research, X-rays have been broadly used to enhance the understanding of material sciences in the past few decades by the interaction of X-rays with materials. γ-Rays can be generated in radioactive materials, which presents fingerprints of elements as well as isotopes, and distinguishing these elements or isotopes needs the detection of the energy-dependent photon counts, or spectroscopy. γ-Ray detection is essential for many homeland security applications such as radiological security, nuclear defense, and so on^6–9^.

Semiconductor-based ionizing radiation detectors work in a similar way with photodetectors either in current or voltage modes, while more details of specific difference are described in Box 1^10,11^. The current advanced radiation detectors working in current mode include silicon (Si), amorphous selenium (α-Se), mercury(II) iodide (HgI_2_), cadmium zinc telluride (Cd_1 − *x*_Zn_*x*_Te), while radiation detectors in voltage mode comprise high-purity germanium (HPGe), Cd_1 − *x*_Zn_*x*_Te, thallium(I) bromide (TlBr), and so on. Besides direct detection strategy, X- and γ-ray detection can also be accomplished in an indirect detection strategy through a combination of scintillators and photodetectors. Scintillators convert high-energy X-rays and γ-rays into ultraviolet–visible (UV–Vis) light, which is detected by weak light sensors such as amorphous Si photodiodes, thin film phototransistor (TFT) arrays, photomultiplier tubes (PMTs), complementary metal-oxide semiconductor (CMOS) detectors, silicon avalanche photodiodes and arrays, or charge-coupled devices (CCDs). The dominating commercial X-ray and γ-ray scintillators include thallium-activated cesium iodide (CsI(Tl)), thallium-activated sodium iodide (NaI(Tl)), and so on. Important performance parameters for scintillators are light yield, light decay time, and environmental stability.

The explosion in the use of higher radiation–dose tests, such as computed tomography (CT) imaging, brings concerns on radiation safety. Over 80 million CT scans are performed in the United States, compared with just 3 million in 1980. High-dose X-ray scan increases the risk of cancer to patient later in life due to the damaging of DNA by ionizing radiation^12^. For example, one-time chest CT scan can have dose equivalent to 2 years accumulation of natural background dose^12^. There is a report showing evidence of radiation-induced cancer risk at a dose above 100 mSv, as well as an estimation of ~2% of the cancers diagnosed could be related to CT performed in the United States in 2007^12^. The high dose needed for CT scanning comes from the low sensitivity of the present radiation detectors, mainly limited by the detection properties of materials. Therefore, high-performance radiation detectors based on new materials are urgently needed.

In the past few years, a new family of halide perovskites with composition of ABX_3_ (where A is CH_3_NH_3_^+^ (MA^+^), HN = CHNH_3_^+^ (FA^+^), or Cs^+^; B is Pb^2+^, Sn^2+^; X is Cl^−^, Br^−^, and I^−^) or A_2_MM′X_6_ (where M is Cu^+^, or Ag^+^, M′ is Bi^3+^, Ga^3+^, or In^3+^) have attracted lots of attention by their excellent performance in solar cells, light-emitting diodes, lasers, and photodetectors during the past few years^13–16^. Halide perovskites are quickly identified to be promising candidate semiconductors for ionizing radiation detection^17–19^. This review exploits the science and application of halide perovskites as a new semiconductor candidate for X-ray imaging and γ-ray spectroscopy. Although there are multiple-stringent requirements on ionizing detector properties, which results in very limited choice on effective materials, halide perovskites show many advantages as promising radiation detector materials, which are discussed in detail in the following sections. There are still outstanding challenges before their commercialization, such as how to stabilize material and interface under large bias in single-photon detectors, which will also be discussed in this review.

Box 1 X- and γ-ray direct detection mechanism X-ray detectors for dosimeter or medical imaging work in current mode, and the photon flux is relatively strong to get enough signal/noise ratio, smooth image, and fast frame rate. Multi-photons come to the semiconductor detector together, and one X-ray photon can be directly converted into many excess free charges within the semiconductors through photoelectric effect process, which are then collected by electrodes under bias as shown in Box 1. In Compton scattering process, the incident photons will be deflected by electrons with a scattering angle with respect to the incident direction, and pass part of their energy to electrons depending on the photon energy and scattering angle. Many charges are generated in these two processes at the same time when attenuation occurs, which will be collected by the electrodes under bias to product signal current. All the attenuated X-ray photons contribute to the average signal current per unit time. The transmitted photon flux intensity (*I*) follows a relationship of $I = I_0 \times e^{ - \alpha \rho l}$, where *I*_0_ is the original photon flux intensity, $\alpha$ is the mass attenuation coefficient, $\rho$ is the materials density, and *l* is the interaction length with materials. The linear attenuation coefficient $\alpha \rho$, also expressed as *μ*_0_, is often used to characterize the stopping power of a material.

While the radiation detectors for photon counters, spectroscopy, and nuclear reaction monitor often work in voltage mode. In contrast to the current mode, the photon flux intensity in voltage mode is relatively weak, and high-energy photons come to a detector one by one. Each photon will generate some electron–hole pairs, and the number of corresponding charge is counted in the signal pulse, the intensity of which is proportional to the photon energy as shown in Box 1, voltage mode. By histogramming the photon energy one after another, a γ-ray photon energy spectrum can be plotted. In order to distinguish the small signal, a charge-sensitive preamplifier is often employed to integrate the signal coming from the detector, thus converting the collected charge into a voltage signal. A quasi-Gaussian output is then performed in a connected shaping amplifier, reshaping the output signal peak to make the transmitted signal better suited to the communication channel, whose intensity is proportional to the photon energy. Finally, a multi-channel analyzer can count the radiation quanta versus energy, and generate X- or γ-ray energy spectrum by recording photons one by one^75,76^

## Development status of conventional radiation detectors

### X-ray dosimeter and imaging detectors by direct conversion

For X-ray imaging detectors, a large sensitivity is needed for high-quality images with large contrast at a given X-ray dose rate^20^, which outputs larger X-ray-induced current^10,21^. In order to minimize the doses applied to the patient while maintaining image quality, one path is to increase the mobility–lifetime (*μτ*) product or increase the applied bias to the detectors^22^.

The *μτ* product, which characterizes carrier drift length, is a fundamental property of a semiconductor material to predict the capability of extracting the charges deep inside the semiconductor. The *μτ* product can be determined by material types as well as defects, which is a parameter closely related to the charge carrier diffusion length *L*_D_ with a relationship of $L_{\mathrm{D}} = \left( {k_{\mathrm{B}}T\mu \tau /e} \right)^{1/2}$, where *k*_B_, *T*, and *e* are the Boltzmann constant, absolute temperature, and elementary charge, respectively. Larger *µτ* product will result in better charge collection efficiency by increasing the mean drift lengths and/or diffusion lengths in the semiconductor bulk. Applying a high bias can enhance the signal intensity; however, it also increases the device noise.

In addition to a strong signal, a small noise is also important for a large signal-to-noise ratio, which is a figure of merit for many types of sensors^18,19,21^. The noise level also determines the weakest X-ray dose a detector can measure. The noise of a radiation detector is generally dominated by dark current, though other types of noise might also be present. While some other figures of merit are also important for a high-quality X-ray image, including spatial resolution, response speed, uniformity, and stability. Spatial resolution is a parameter used to characterize image sharpness, which can be quantitatively assessed by the modulation transfer function (MTF)^23^. Linear dynamic range (LDR) is another important parameter for an X-ray detector to show the responsible range of X-ray dose rate under which sensitivity is a constant. Large LDR will ensure that the detector gives an accurate measurement of X-ray dose rate under a large variation range. Moreover, the X-ray detector needs to have a fast response speed to reduce the exposure time of X-ray irradiation, and also give high frame rate for capturing videos. Frame rate is the inverse of the time needed for the CCD to acquire an image and then completely read an image out, which is not only related to device response time but also the read-out circuitry. A uniform performance of the pixels among large area is also crucial for a high-quality X-ray imager, though it can be partially made-up with imaging processing.

### Device performance of conventional X-ray detectors

Table 1 summarizes the detailed performance of the current X-ray detectors. They are made by Si, α-Se, HgI_2_, Cd_1 − *x*_Zn_*x*_Te, and so on. Since Si has low stopping power to X-ray, it is mainly applied in portable X-ray fluorescence spectrometry for soft X-ray detection, especially for photon energy smaller than 10 keV to detect the fingerprints of trace elements as well as isotopes of different bulk materials^24^. Since α-Se has over ten times stronger stopping power than Si in hard X-ray range^25,26^, it is the most common semiconductor material for X-ray imaging in direct conversion mode, and it has dominated the direct conversion X-ray imaging market for decades^10^. Although the cost of α-Se raw material is only ~$0.8 per g, the main flat-panel detector cost comes from thick film deposition process by conventional vacuum thermal deposition. HgI_2_ and Cd_1 − *x*_Zn_*x*_Te (*x* is <20%, denoted as CZT) are both promising X-ray detectors. Their sensitivities are good as shown in Table 1, but the performance of HgI_2_ detector is mainly limited by the large leakage current. While there are also challenges in integrating CZT with read-out circuitry due to the too high temperature that required for high-quality CZT crystal growth.

**Table 1:** Performance of conventional X-ray detectors for medical imaging

	Atomic number	Applied electric field (V μm^−1^)	*μτ* product (cm^2^ V^−1^)	Sensitivity (μC Gy^−1^_air_ cm^−2^)	Spatial resolution (lp mm^−1^)	Lowest detectable dose rate (μGy_air_ s^−1^)	Refs
Si^a^	14	0.5	>1	8	4.5	<8300	^24, 26^
a-Se^a^	34	10	10^−7^	20	~15	5.5	^21, 70^
HgI_2_^a^	53,80	10	10^−4^	1600	3.93	10	^84^
CZT^a^	48, 52	0.1–1	0.01	318	10	50	^85, 86^
MAPbBr_3_^a^	35, 82	0.05	0.012	2.1 × 10^4^	10	0.039	^18, 66^
MAPbI_3_^a^	53, 82	0.24	0.010	1.1 × 10^4^	3	<5000	^64^
Cs_2_AgBiBr_6_^a^	47, 53, 55, 83	0.025	0.0060	105	—	0.060	^47^
CsI(Tl)^b^	53, 55, 81	—	—	5370	10	0.18	^27, 28^
GOS(Tb)^b^	64, 65	—	—	7.3	4.75	—	^29^

^a^The direct X-ray detection by semiconductor

^b^The indirect X-ray detection by scintillator

The current state-of-the-art CsI(Tl) scintillator detectors with CMOS sensor have pixel resolution around 50μm, or 10 lpmm^−1^ (ref. ^27^), and corresponding X-ray sensitivity^28^ reaches 5370 μC Gy^−1^air cm^−2^. A recent reported scintillator made by terbium-doped gadolinium oxysulfide (GOS:Tb) has been employed for X-ray imaging with spatial resolution of 4.75 lp mm^−1^ (ref. ^29^). Although the resolution is not high and the sensitivity is only about 7.3μCGy^−1^air cm^−2^, GOS:Tb scintillator are still attractive for its low-cost fabrication from solution.

### γ-Ray spectroscopy detectors

As for the γ-ray counting for energy spectrum application, the most important figure of merit is the photopeak energy resolution, which is defined by the ratio of full-width at half-maximum value of the photopeak and its corresponding photon energy. The semiconductor detectors for γ-ray spectrum acquisition actually have been developed for several decades, while there are still only a handful of semiconductors that can be successfully applied for this purpose with satisfying performance^30^, since γ-ray detection imposes multiple-stringent requirements on semiconductor materials. Due to the much higher energy of γ-ray than X-ray, it needs materials to possess large atomic number (*Z*) for strong stopping power^31^; large electron and hole *µτE*′*’*product (or Schubweg distance) for efficient charge collection, where *E*′ is the applied electric field; a low density of trap states to avoid charge trapping or recombination during charge collection for single event analysis; again, a large bulk resistivity over 10^9^ Ωcm is paramount for high signal/noise ratio.

Table 2 lists several figures of merit of typical γ-ray semiconductor detectors that can impact the energy spectrum resolution. HPGe-based spectrum detectors now gives the best energy resolution of 0.3%^32,33^, while it requires cryogenic cooling to suppress its dark current due to its small bandgap of 0.66 eV. CZT materials have a larger bandgap of over 1.6 eV, and its large Z elements ensure a strong stopping power. A significant amount of efforts have been devoted to increase the bulk resistivity to around 10^9^ to 10^10^ Ω cm by crystal growth. The state-of-the-art CZT detectors achieved an energy resolution of 0.5%, which operates at room temperature^34,35^. TlBr is another semiconductor that makes detectors with energy resolution of about 1% at room temperature, because of its large stopping power (four to five times stronger than CZT), high resistivity of over 10^11^ Ω cm, and large bandgap of 2.68 eV, although the best reported *μτ* product of this material is still one order of magnitude smaller than CZT^36^, resulting in a very good energy resolution of 1%. Notably, high-quality CZT materials are also not broadly commercially available, and now CZT single crystals cost about $3000 per cm^3^. While it is possible that the cost of CZT single crystals can be dramatically reduced by scaling up the manufacturing, industry is pushed back by the small demand of CZT materials in the radiation detection market, in strong contrast to silicon.

**Table 2:** Detailed γ-ray detector for energy spectrum

	Atomic number	Linear attenuation coefficients to 662 keV^a^ (cm^−1^)	Bandgap (eV)	Bulk resistivity (Ω cm)	*μτ* product (cm^2^ V^−1^)	Energy resolution (%)	Refs
HPGe^b^	32	0.01	0.67	10^2^–10^3^	>1	0.2	^33, 87^
CZT^b^	48, 52	0.05	1.5–1.6	10^10^	0.004–0.01	0.5	^34, 35^
TlBr^b^	35, 81	0.24	2.68	10^11^–10^12^	0.0005	1	^36^
Halide perovskite^b^	35, 53, 55, 82, 83	0.09	1.5–3.1	10^7^–10^10^	0.001–0.01	3.9	^46, 48, 72, 81^
NaI(Tl)^c^	53, 81	0.01	3.0	—	—	5.6	^2^

^a^ Only photoelectric attenuation process is considered to calculate the attenuation coefficients

^b^The direct γ-ray detection by semiconductor

^c^The indirect γ-ray detection by scintillator

Similar to X-ray imaging, γ-spectrum can be also collected by a combination of scintillator and a sensitive pixel photodetector. Typical scintillators for γ-application are made of NaI(Tl) crystals and CsI(Tl) crystals. NaI(Tl) crystal emits 415 nm light with efficiency of 11.3%, comparable to the CsI(Tl). The emitting light will be detected by a PMT detector to record light intensity, which is proportional to the γ-ray energy. Recent technology is moving toward γ-detector array so that spectrum can be quickly collected by multiple detectors, which however needs a really good uniformity of performance among all these γ-ray pixels.

## Halide perovskite materials emerge as promising candidates for radiation detectors

Right after the demonstration of efficient solar cell application, halide perovskites were shown to be good photodetector materials due to the large light absorption coefficient, tunable bandgap, large mobility, and long carrier recombination lifetime, even in the form of polycrystalline films made from solution^37–40^. All these properties are actually also desired for radiation detection. In this section, we review the properties of perovskites, which make them a new generation of radiation detection materials.

### Strong stopping power

**Fig. 1:**
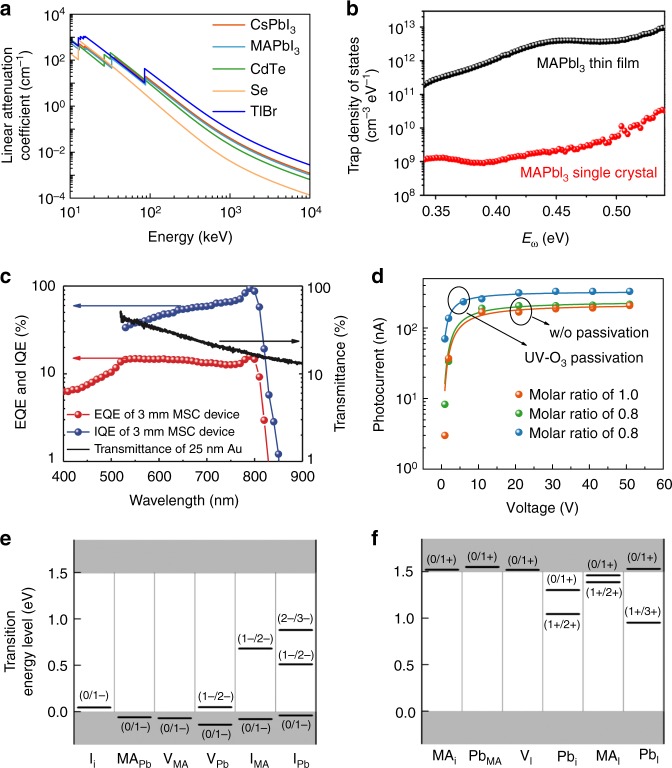
Electronic properties of halide perovskites. **a** Linear attenuation coefficient of MAPbI_3_, MAPbBr_3_, CdTe, Se, and TlBr versus photons energy. **b** Trap density of states of the MAPbI_3_ single crystal (red dots) and the thin film (black dots) measured by thermal admittance spectroscopy at room temperature. **c** External quantum efficiency (EQE) and internal quantum efficiency (IQE) spectra of a 3-mm-thick MAPbI_3_ single crystal along with the transmittance of 25-nm-thick gold electrode. **d** The *µτ* product of MAPbBr_3_ crystal devices with different raw materials feeding ratio and surface passivation procedure. The transition energy levels of **e** intrinsic acceptors and **f** intrinsic donors in CH_3_NH_3_PbI_3_ perovskite. **b**, **c** are adapted with permission from ref. ^43^, AAAS. **d** is adapted with permission from ref. ^18^, Springer Nature. **e**, **f** are adapted with permission from ref. ^49^, American Institute of Physics

In halide perovskites, the atomic number of Cs^+^, Pb^2+^, Ag^+^, Bi^3+^, Sn^2+^, I^−^, and Br^−^ ions are as high as 55, 82, 47, 83, 50, 53, and 35, respectively. Some of the atomic numbers are even larger than those of CZT as shown in Table 2. The typical MAPbI_3_ halide perovskite has a density of around 4 g/cm^3^, providing a large linear attenuation coefficient of 10 cm^−1^ to 100 keV X-rays, which is comparable to that of CZT material as shown in Fig. 1a and Table 2. Inorganic halide perovskite CsPbI_3_ has a larger linear attenuation coefficient of 14 cm^−1^. The capability to form double perovskites, such as Cs_2_AgBiI_6_ and Cs_2_AgBiBr_6_, allows the incorporation of many other high *Z* elements into the perovskite structure.

### Large mu–tau product

Halide perovskites were first applied in dye-sensitized solar cell structure as dye on mesoporous TiO_2_ surface^41^, while researchers later found that halide perovskite solar cells with planar heterojunction structure works well due to its long charge carrier diffusion length of over 1000nm^42^, corresponding to a mu–tau(*µτ*) product of 3.9 × 10^−7^ cm^2^ V^−1^. This result was confirmed by Yakunin et al.’s^17^ work using perovskite polycrystalline films for X-ray detectors. Perovskite single crystal presents much lower trap density by removing the grain boundaries of polycrystals as shown in Fig. 1b as well as improved crystallinity. The internal quantum efficiency (IQE) spectrum in Fig. 1c shows that the charge collection efficiency of a 3-mm-thick MAPbI_3_ single crystal is approaching 100%, indicating an extremely long charge carrier diffusion length^43^. The mobility and charge recombination lifetime measurement results show a large *µτ* product of 1.2 × 10^−2^ cm^2^ V^−1^, corresponding to a long diffusion length of 175 µm as the low limit under strong illumination, which is consistent with photoconductivity measurement showing large *µτ* product of MAPbBr_3_ perovskite single crystal^18^, as shown in Fig. 1d. The *µτ* products of MAPbI_3_ and MAPbBr_3_ single crystals are comparable to that of CZT crystals, and are much larger than TlBr at room temperature, as shown in Table 2.

### Large bulk resistivity

The bulk resistivity of the semiconductors is another important figure of merit in radiation detectors for small dark current and noise. One challenge in halide perovskite field is the difficulty in doping them intentionally, although this makes them attractive for radiation detection. Nevertheless, these materials are still subjected to self-doping. Halide perovskites showing excellent photovoltaic performance are mostly intrinsic or weakly *p*-type^44^. Replacing partial or all I^−^ ions with Br^−^ ions or Cl^−^ ions can reduce the intrinsic charge carrier concentration due to enlarged bandgap. Typical MAPbI_3_ perovskite has a resistivity of around 10^7^Ω cm at room temperature^45^, and the resistivity of MAPbBr_3_ perovskite can be normally ten-folds higher^46^. These resistivity are still much smaller than those calculated from intrinsic charge concentration, indicating an unintentional doping in these materials, which was reported^44^. While the resistivity of inorganic halide perovskites can reach 10^10^ Ω cm, satisfying the requirement for γ-ray spectroscopy, but the mobility or *µτ* product is much lower than CZT materials^47,48^ (See Table 2).

### Radiation hardness

One unique property of halide perovskites is its relatively better tolerance to defects than other semiconductor materials. First-principle calculation shows that the deep charge traps in MAPbI_3_ such as I_Pb_, I_MA_, Pb_i_, and Pb_I_ have too large formation energy to form. Most point defects either in the conduction or valence bands are shallow traps, such as MA_i_, V_Pb_, MA_Pb_, I_i_, V_I_, and V_MA_^49^ (see Figs 1e, f). The defect tolerance may explain the reported long electron–hole diffusion length in the single crystalline materials^42,43^. The defect tolerance also gives rise to the radiation damage tolerance of these materials^50–52^.

Lang et al.^53^ reported the stability of MAPbI_3_ solar cells under proton irradiation. A reference device with initial power conversion efficiency (PCE) of 12.1% has only 20% drop in current density, but with no voltage and fill factor loss after irradiation with 68 MeV protons with dose up to 1.02 × 10^13^ p cm^−2^. It is also encouraging to find that the device has self-healing capability, that is, the current density of the device recovered to its initial value after shelf storage for 10 days^53^. Chen et al.^54^ found that the Frenkel defects were generated by applying bias on perovskite, which may be related to the current density loss under irradiation, since the built-in electric field may also cause Frenkel defects. Self-annihilation of Frenkel defects is also confirmed when releasing the electric field, which is in consistent with the observed self-healing effect. Yang et al.^55^ also tested the stability of perovskite solar cells to γ-ray irradiation. The PCE barely drops after over 1500 h continuous γ-ray irradiation with total exposed dose of 23 kGy, while glass is significantly colored by γ-ray irradiation^55^. In striking contrast, CZT detectors lose their performance severely after total 30 kGy γ-ray exposure^56^, indicating the good γ-ray hardness of perovskite materials.

### Low-cost raw materials and crystal growth

The raw materials for halide perovskite are nature abundant and low cost. The total material cost for 1 cm^3^ perovskite single crystal is about $2.65 to $3.65 based on raw material prices from suppliers such as Sigma-Aldrich. Considering the material cost generally reduces by 5 to 10 times after scaling up the production, we estimate that material cost of halide perovskite single crystals is <$0.3 per cm^3^, which is 3 to 4 orders of magnitude cheaper than CZT crystals. The device fabrication cost by single crystal growth or polycrystalline films deposition process is also low, because they are all done by low-cost solution processes. Large and high-quality perovskite single crystals can be grown from low-cost solution processes^57–60^ at relatively low temperature (lower than 150 °C) compared to traditional radiation detector crystals such as HPGe and CZT, which are often grown at temperatures around the materials’ melting points.

**Fig. 2:**
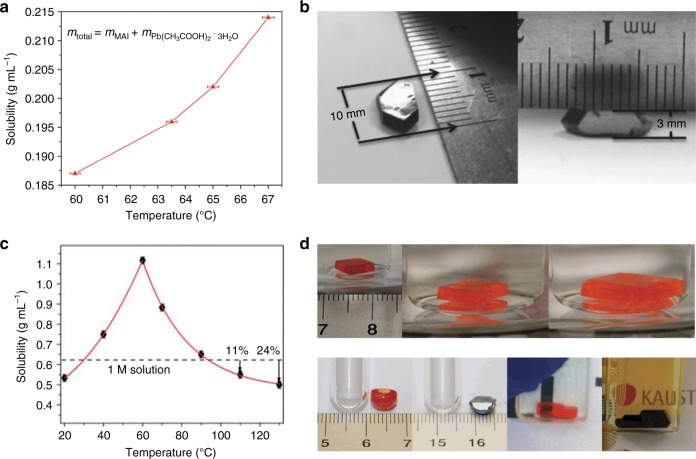
Perovskite single crystals growth. **a** Solubility of MAPbI_3_ perovskite versus temperature in HI acid. **b** Photos of as-grown single crystal by top-seeded solution growth method. **c** Reverse solubility of MAPbI_3_ perovskite versus temperature in γ-butyrolactone (GBL). **d** Photos of as-grown halide perovskite single crystal by inverse temperature method. **a** is adapted with permission from ref. ^57^, RSC Publishing. **b** is adapted with permission from ref. ^43^, AAAS. **c** is adapted with permission from ref. ^58^, American Institute of Physics. **d** is adapted with permission from ref. ^59^, Springer Nature

In hydrohalic acid, halide perovskites solubility often increases at higher temperatures ^57^ as shown in Fig. 2a, and single crystals with several millimeter size can be grown by gradually reducing the solution temperature as shown in Fig. 2b. While in some organic solvents such as dimethylformamide and γ-butyrolactone (GBL), halide perovskite has a reduced solubility at higher temperature (Fig. 2c), in striking contrast to many other materials. It was argued that the inverse solubility was caused by the protons as a result of solvent degradation under high temperature^61^, while exception to this rule can be easily named. The single crystal from such solvents can be grown by increasing the temperature from saturated solution^58^, and high-quality halide perovskite with dimension size over several millimeters can be obtained at a fast rate of 0.1–0.2 cm h^−1^ as illustrated in Fig. 2d ^59^. The measured hole carrier mobility of MAPbI_3_ single crystal grown from GBL solvent is about 67 cm^2^ V^−1^ s^−1^, over two times smaller than mobility of 164cm^2^ V^−1^ s^−1^ of MAPbI_3_ single crystal grown from HI solvent^43^. This may be caused by the better quality of crystals grown from hydrohalic acid, since the crystal growth speed in hydrohalic acid is as slow as 0.2 mm per day. In addition, an anti-solvent approach has also been employed to grow perovskite single crystals from mixture solvents, which may give uniform growth rate of single crystals at room temperature^62–64^.

## Advance in the development of perovskite X-ray imaging detectors

**Fig. 3:**
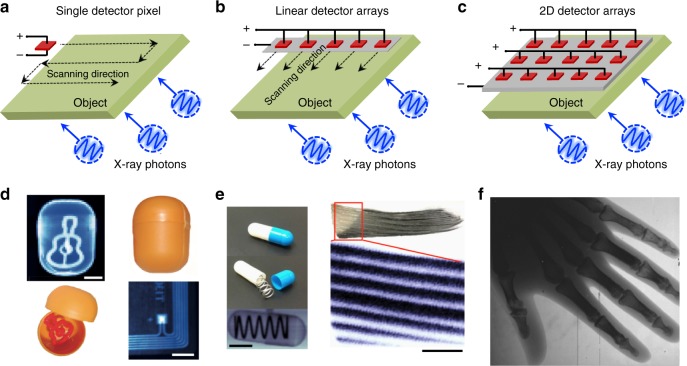
X-ray imaging by single pixel, linear arrays, and two-dimensional (2D) arrays scanning. **a–c** Scheme of three different X-ray imaging processes: **a** single pixel scanning, **b** linear detector array (LDA) scanning, **c** 2D detector arrays scanning. **d** Optical and X-ray images of a plastic toy and an electronic key card. **e** Optical and X-ray images of an encapsulated metallic spring as well as a portion of a fish caudal fin (top, right) and X-ray image of a section of it (bottom, right). **f** A hand phantom X-ray image obtained from a muon piston calorimeter (MPC) detector (using 100kVp and 5mGy_air_ s^−1^ for 5 ms exposure, resulting in a dose of 25μGy_air_ and a bias voltage of 50 V). Scale bars in **d** are 10 mm and the ones in **e** are 5 mm. **d** (ref. ^17^), **e** (ref. ^66^), and **f** (ref. ^64^) are all adapted with permission from Springer Nature

The first perovskite radiation detector made by MAPbI_3_ single crystal showed response to high-energy γ-ray photons under zero bias^43^. The device worked in γ-voltaic mode with a quantum efficiency of 4% under high flux γ-ray. Later polycrystalline perovskite films made by spray coating were demonstrated to have good X-ray response with imaging capability by moving a single pixel detector along X–Y directions^17^. By scanning the signal current induced by transmitted X-ray photons, X-ray images of plastic target with clear inner scene were observed, as illustrated in Fig. 3a–d. The imaging time using single pixel detectors is very long, since it takes a lot of time to move the detector or the object along the X–Y plane. Nevertheless, it is still a good conceptual demonstration for perovskite materials for X-ray imaging application. Commercial X-ray detector products for imaging often employ linear detector arrays (LDAs), or, more frequently, flat panel arrays for fast detection. LDAs composed of individual X-ray-sensitive materials are arranged in a line as linear pixels as shown in Fig. 3b, which can scan along one direction to finish imaging (Fig. 3e). Flat panel arrays (Fig. 3c), which are often constructed by integrating X-ray-sensitive semiconductors on application-specific integrated circuits like CMOS read-out circuitry, can be employed to map an object by detecting the spatially dependent transmitted photon flux^10^. In this section, we review advancements in the development of perovskite single crystals and polycrystalline films for X-ray detection in the form of single pixel, LDAs, and two-dimensional (2D) detector arrays.

### Single pixel perovskite detectors

Due to the large atomic number and material density of halide perovskite, the linear attenuation coefficient of MAPbI_3_ to 120 keV X-rays is about 10 times stronger than α-Se. Nevertheless, MAPbBr_3_ with slightly weaker X-ray stopping power has been more often used for X-ray detection, because of many advantages including simple single crystal growth, cubic crystal shape, large bandgap, easily crystal quality inspection due to its transparency in visible light range, and less ion migration. One issue frequently encountered is that the MAPbBr_3_ single crystals are not transparent with visible defects, which can be identified by the naked eye. Highly transparent MAPbBr_3_ single crystals are grown using precursor solution with non-stoichiometry precursor ratio. Since the PbBr_2_ has less solubility and precipitates faster than MABr in perovskite solution, a PbBr_2_/MABr feeding ratio of 0.8 can get equal precursor precipitating speed, which allows the growth of high-quality single crystals. The *µτ* product of such MAPbBr_3_ single crystals reached 1.4 × 10^−2^ cm^2 ^V^−1^ (ref. ^18^). Moreover, Cl^−^ ions alloying can further improve the hole mobility to 560 cm^2 ^V^−1^ s^−1^ (ref. ^46^), which increased *µτ* product to 1.8 × 10^−2^ cm^2 ^V^−1^. In addition to *µτ* product, the surface charge recombination rate (*s*) at perovskite/electrode interface also determines the signal intensity of an X-ray detector, and its dependence can be described by modified Hecht’s equation:

1 $$I = \frac{{I_0\mu \tau V}}{{L^2}}\frac{{1 - {\mathrm{exp}}( - \frac{{L^2}}{{\mu \tau V}})}}{{1 + \frac{L}{V}\frac{s}{\mu }}},$$

where *I* is the measured photocurrent, *I*_0_ is the saturated photocurrent, *L* is the thickness, and *V* is the applied bias. Clearly, a smaller *s* can also increase photocurrent. One attractive property of MAPbBr_3_ in this context lies its automatic surface passivation by oxygen or moisture in air. The charge radiative recombination rate on the surface of MAPbBr_3_ single crystals varied by over two orders of magnitude upon physisorption or desorption of oxygen and water molecules^65^. Full physisorption of oxygen on MAPbBr_3_ surface resulted in a low surface recombination velocity of 4 cm s^−1^ in this material, even better than that well-passivated single crystalline silicon. In real operational devices, UV–ozone treatment of MAPbBr_3_ surface is a good strategy to retain the oxygen passivation effect, which successfully suppressed the surface recombination and resulted in a high X-ray sensitivity of 80μCGy^−1^air cm^−2^ for the detector under a small bias of 0.1 V^18^. This sensitivity is already four times higher than α-Se X-ray detectors as shown in Table 1^21^.

After demonstrating the superior detection properties of MAPbBr_3_ single crystals, a further step was taken to integrate them onto silicon, glass and other metallic substrates, which is touchstone to evaluate integration capability of perovskites with CMOS read-out circuitry. MAPbBr_3_ can be grown in solution at low temperatures below 120 °C, which is compatible with CMOS read-out circuitry. Wei et al.^66^ used a small-molecule of (3-aminopropyl)triethoxysilane layer, which has a NH_3_Br terminate on one end and Si-O bond on the other end, to mechanically and electrically connect the MAPbBr_3_ single crystals with any surface that can bind with Si-O bond^66^. The dipole of this bonding molecule at the interface of perovskite and Si also suppress the noise current while maintaining the signal current, which allows the device to operate under a larger optimal bias of −7V. The large gain at larger bias made the Si-integrated MAPbBr_3_ single crystal detectors to have a much larger sensitivity of 2.1 × 10^4^ μCGy^−1^air cm^−2^ under 8 keV soft X-ray. The lowest detectable dose rate was also further reduced down to 36nGy_air_ s^−1^, much better than the α-Se detectors as shown in Table 1. Cs_2_AgBiBr_6_ single crystals have large resistivity over 10^10^ Ω cm despite small *μτ* product, which results in a detectable lowest X-ray dose of 59.7nGy_air_ s^−1^.

In addition to single crystals, polycrystalline perovskite bulk materials are formed by high-pressure sintering process from perovskite powders for X-ray detectors^63^. A polycrystalline perovskite wafer with diameter of 0.5 in. and thickness of 1 mm was demonstrated. Although the *μτ* product of the polycrystalline perovskite wafer was only 2 × 10^−4^ cm^2^ V^−1^, about two orders of magnitude lower than that of the single crystals, a reasonably large sensitivity of 2527μCGy^−1^air cm^−2^ was obtained under a relatively large electric field of 0.2 Vμm^−1^ (see Table 1). The challenge will be how to integrate them into CMOS read-out circuitry, because the high-pressure or high temperature process is often not compatible with CMOS technology.

### Linear detector arrays

LDA scanning is more convenient than 2D arrays imaging in some cases where very large area X-ray imaging needs to be performed by portable detectors like cargo inspection. The first perovskite LDA was demonstrated by depositing ten pixel electrodes in a row on a MAPbBr_3_ single crystal with a pixel electrode size of 200 μm and a pitch of 400 μm. The LDA devices by MAPbBr_3_ single crystal was demonstrated to possess good X-ray imaging capability at a very low dose rate of 36 nGy_air_ s^−1^ (ref. ^66^). Dose–area product (DAP) rate is a figure of merit used to compare absorbed doses in real medical imaging^67^. DAP rate of around 2.5 Gy_air_ cm^2^ h^−1^ is estimated for perovskite detector in interventional cardiology with X-rays continuously on for capturing an image (entrance area of 100 cm^2^) of the human body, at least an order of magnitude better than that offered by state-of-the-art commercial medical radiographic systems^68,69^. The resolution of X-ray image reached 10 lp mm^−1^, which is comparable to those of the best CZT X-ray detectors and CsI(Tl) scintillator detectors as shown in Table 1, and is still lower than α-Se flat panel imagers^70^. In this case, the lateral diffusion of carriers seemed not to affect the resolution of these detector arrays, despite that the crystals was still thick, maybe due to the relatively large electric field applied.

### 2D detector arrays

Low temperature growth of perovskite single crystals makes it compatible with the CMOS read-out circuitry, and promising for high-sensitivity 2D X-ray detector arrays. The first 2D X-ray detector array was made of perovskite polycrystalline films^64^. Similar to α-Se, polycrystalline perovskite films are actually attractive options for flat panel imagers, because they can be deposited directly on read-out circuitry with existing deposition techniques.

Doctor blading has been recently established to deposit halide perovskite films for solar cell modules with area close to 225 cm^2^ with module PCE of ~15%^71^. Spray coating is another mature technique to deposit thick films, and perovskite X-ray pixel detectors were first demonstrated in 2015 by employing spray-coated MAPbI_3_ polycrystalline films with thickness of 60 μm, which is thick enough to detect soft 8 keV X-rays^17^. Fullerene layers are often employed in these cases to block the pin holes to suppress leakage current^37^, and also passivate the surface charge traps on perovskites^32^. Recently, Kim et al.^64^ doctor-bladed 830-μm-thick MAPbI_3_ films on a 10 cm × 10 cm large TFT substrate^64^. Under irradiation from a 100kVp bremsstrahlung X-ray source, the 2D detector arrays presented good X-ray response under 50 V bias, showing clear X-ray image of a hand with bones observed inside in Fig. 3f. The image was acquired with 5 ms exposure of X-rays with dose rate of 5mGy_air_ s^−1^, resulting in an exposed total dose of 25μGy_air_. The pixel size was around 70μm×70μm, while the resolution was only 3lpmm^−1^. This indicates that the resolution in these 2D arrays is still limited by the lateral charge diffusion among the crystal grains underneath neighboring pixel arrays, rather than the pixel size.

## Perovskite γ-ray detectors for photon energy spectrum

**Fig. 4:**
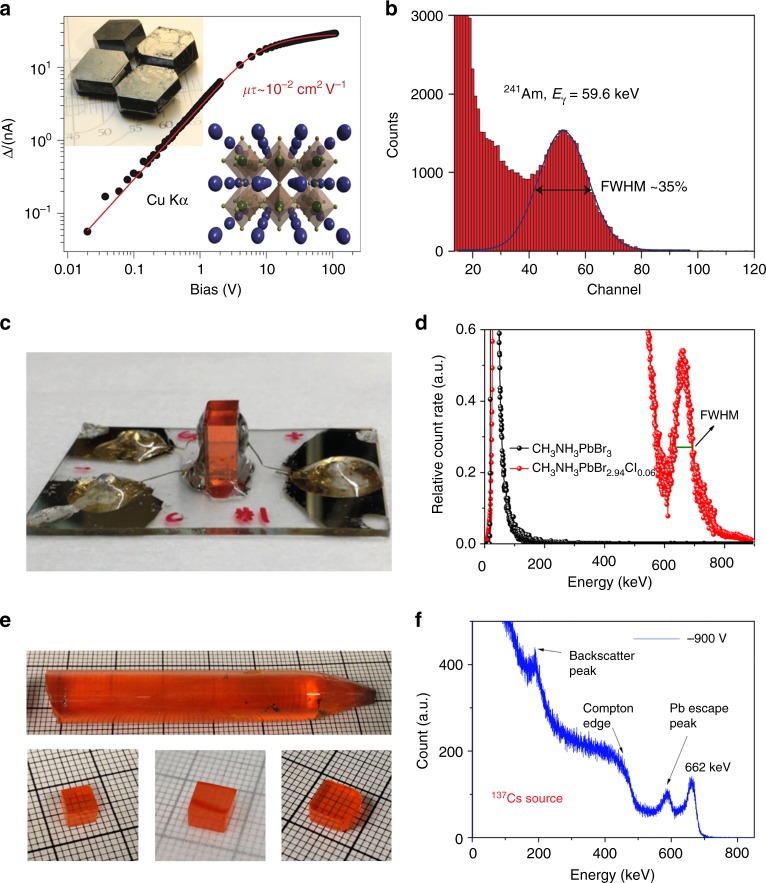
γ-Ray spectrum detection by halide perovskites. **a** The bias dependence of the photocurrent generated by Cu Kα X-ray in a MAPbI_3_ single crystal (SC); the red line indicates a fit with the Hecht model. Top inset: Photograph of typical MAPbI_3_ perovskite SCs grown from a non-aqueous method. Bottom inset: Schematic of the three-dimensional interconnection of PbI_6_ octahedra in a perovskite lattice (green, Pb; yellow, I; blue, MA). **b** Energy-resolved spectrum of ^241^Am recorded with a FAPbI_3_ SC. **c** Side view of a CH_3_NH_3_PbBr_2.94_Cl_0.06_ SC detector, and electrode sides were encapsulated with epoxy. **d** Enlarged photopeak region of the ^137^Cs energy spectrum obtained by CH_3_NH_3_PbBr_2.94_Cl_0.06_ and CH_3_NH_3_PbBr_3_ SC detectors. **e** As-grown CsPbBr_3_ SC ingot with a diameter of 11 mm, and the SC wafers with different sizes. **f** Energy-resolved spectrum of ^137^Cs γ-ray source with the characteristic energy of 662 keV obtained by a CsPbBr_3_ detector. **a**, **b** (ref. ^72^), **c**, **d** (ref. ^46^), and **e**, **f** (ref. ^48^) are all adapted with permission from Springer Nature

In contrast to X-rays, γ-rays have much higher energy and stronger penetrating capability. Fortunately, perovskite material has a strong stopping power to γ-rays with linear attenuation coefficient of 0.09 cm^−1^ at 662 keV (photon energy of ^137^Cs source), nearly two times larger than that of CdTe material^46^ (Fig. 1a and Table 2), and high-quality perovskite single crystals with centimeter size can be also easily grown from solution, which together enable sufficient stopping of γ-ray photons. Yakunin et al.^72^ firstly reported perovskite single crystal detectors toward γ-ray detection made by formamidinium lead triiodide (FAPbI_3_) single crystals. The crystals are shown in the inset of Fig. 4a, which have a large *µτ* product of 1 × 10^−2^ cm^2^ V^−1^, and the devices showing good response to 59.6 keV ^241^Am γ-ray photons at room temperature (Fig. 4b). However, the black phase of FAPbI_3_ single crystal is not thermodynamically stable at room temperature. Higher energy γ-ray spectrum cannot be acquired in this case, since a larger crystal and larger bulk resistivity for the crystal are needed for stronger stopping power and smaller dark current, respectively.

Good γ-ray detectors should avoid the polarization effect, which describes the phenomenon of continuous dark current drift and associated degradation of detector energy spectrum resolution^73^. The polarization effect is related to the ion migration in many γ-ray detecting semiconductors materials studied so far^74^. The dark current stability and energy spectrum stability of device can be monitored to evaluate the degree of the polarization effect, which is also a very common method in traditional radiation detectors, such as CZT and TlBr single crystals^73,75,76^. Improving the bulk crystal quality and electrode contact can partially suppress the polarization effect at room temperature, although low temperature will also freeze the mobile ions^77^. Ion migration has been confirmed in halide perovskite materials, and the grain boundaries in polycrystalline films are shown to be the dominating ion migration channels^78^. Nevertheless, polarization effect (or ion migration) can still occur in single crystals, depending on crystal quality, crystal composition, and applied electric field. For example, ion migration is often more notable in I^−^-based perovskite than Br^−^-based perovskites. Nazarenko et al. improved the FAPbI_3_ single crystal detector performance by alloying Cs^+^ ions and Br^−^ ions into bulk crystals to get mixture halide perovskite single crystal with composition of Cs_*x*_FA_1 − *x*_PbI_3 − *y*_Br_*y*_ (*x* = 0–0.1, *y* = 0–0.6)^79^. Since Cs^+^ ions and Br^−^ ions can partially stabilize the black phase of FAPbI_3_ single crystal, these have less ion migration compared to FA^+^ ions and I^−^ ions at room temperature, respectively^80–82^. However, despite that the Cs_*x*_FA_1 − *x*_PbI_3 − *y*_Br_*y*_ mixture perovskite single crystal showed *µτ* product of 0.12 cm^2^ V^−1^, neither the photopeak or the Compton edge were sharp or clear in the obtained spectrum^79^. No reason was given in this work, but we suspect the low bulk resistivity and large device noise/dark current in this crystals lead to a low signal-to-noise ratio.

Dopant compensation is a good strategy to increase the resistivity of MAPbBr_3_ by alloying *n*-type MAPbCl_3_ into *p*-type MAPbBr_3_ to reduce its self-doping level^46^. The MAPbBr_3 − *x*_Cl_*x*_ perovskite single crystal gradually changed from *p*-type to *n*-type as increased Cl^−^ ions amount. MAPbBr_2.94_Cl_0.06_ perovskite single crystal is almost intrinsic and has the lowest charge carrier concentration among all the alloys. The alloying technique also increased the *µτ* product from 1.2×10^−2^ to 1.8×10^−2^ cm^2^ V^−1^. By suppressing the crystal surface/edge leakage current with a guard ring electrode^83^ (Fig. 4c), a ten-fold improved bulk resistivity to 3.6 × 10^9^ Ω cm is revealed for the MAPbBr_2.94_Cl_0.06_ perovskite single crystals^46^. ^137^Cs γ-ray energy spectrum with all features was then acquired at room temperature, benefiting from the high signal-to-noise ratio. The best photopeak energy resolution reached 6.5% as shown in Fig. 4d, as the first demonstration of γ-ray energy spectrum collection capability by halide perovskite single crystals^46^. Recently, He and co-workers improved the quality of CsPbBr_3_ single crystals grown from melt (Fig. 4e) by slowing down the crystal growth speed with a two-stage cooling process, first 20 K h^−1^ for 20 h and then 5 K h^−1^ for another 35 h, which is critical parameter for crystal quality grown by high temperature Bridgman method^48^. Asymmetry electrodes of gold (Au) and gallium (Ga) serving as working electrodes on opposite surfaces of perovskite single crystals not only efficiently collect charges due to their work function difference^43^, the Schottky junction also suppressed device noise^48^. The crystals maintained a good *µτ* product of 1.34 × 10^−3^ cm^2^ V^−1^. ^137^Cs γ-ray spectrum with 662 keV photopeak was obtained with energy resolution of 3.9% using a thin crystal as shown in Fig. 4f, comparable to, or even better than, the commercial NaI(Tl) scintillator (See Table 2).

## Challenges and outlook

Halide perovskites have been demonstrated as a new generation of room temperature radiation detection materials in the past 5 years. Efforts are still needed to improve the sensitivity of X-ray detectors for imaging, because the lowest detectable dose rate by the best reported perovskite detectors is still much higher than the average background irradiation of 0.1nGy_air_ s^−1^ in United States. Although increasing the working bias can enhance charge extraction in the detectors, it also increases noise. The dark current density of halide perovskite X-ray detectors is still higher than that of the commercial α-Se detectors. Therefore, the resistivity of perovskites needs further improvement based on new material design and composition. The background charge carrier concentration is still dominated by the self-doping effect in halide perovskites, which limits the lowest detectable X-ray dose rate. Further improvement can be expected if deep mechanism of self-doping is understood. Although these hybrid perovskite materials are more prone to degradation after exposure to heat or moisture than conventional detectors, it remains to be determined whether the environmental stability would impact the X-ray detectors, because perovskites will be encapsulated by X-ray penetration materials.

For spectroscopy applications, the state-of-the-art room temperature γ-ray detectors are made of CZT single crystals, which can achieve an energy resolution of around 0.5%, although it takes several decades for them to reach this high resolution. The performance of halide perovskite γ-ray detectors is far from this level. The relatively lower bulk resistivity is among one of the challenges to be addressed. The size of high-quality single crystals is not large yet, though there should be no fundamental limit for achieving larger size. The competition between charge collection and charge recombination in these big crystals is not understood yet. Long-term continuous operation of halide perovskite devices under large field to detect X-rays and γ-rays is still a major concern, since the polarization effect may be still notable at room temperature. Designing a good perovskite X-ray imager or spectrometer should consider all the required material properties as discussed that lead to a high signal-to-noise ratio and fast response. Considering the nature-abundant and low-cost raw materials with all the exciting discoveries in such a short time, halide perovskites still hold a big promise to compete with traditional ionizing radiation detection materials.
